# A Re-Evaluation of the Causes of Deformation in 1Cr-1Mo-0.25V Steel for Turbine Rotors and Shafts Based on iso-Thermal Plots of the Wilshire Equation and the Modelling of Batch to Batch Variation

**DOI:** 10.3390/ma10060575

**Published:** 2017-05-24

**Authors:** Mark Evans

**Affiliations:** College of Engineering, Swansea University, Fabian Way, Crymlyn Burrows, Wales SA1 8EN, UK; m.evans@swansea.ac.uk; Tel.: +44-1792295748

**Keywords:** creep, Wilshire equations, iso-thermal plots, prediction bands

## Abstract

The aims of this paper were to: (a) demonstrate how iso-thermal plots of the Wilshire equation can be used to identify the correct structure of this equation (which in turn enables a meaningful description of the creep mechanism involved in deformation to be made); and (b) show how a generalized specification of batch to batch variation could produce less conservative predictions of the time to failure associated with a given degree of risk. Such predictions were obtained using maximum likelihood estimation of the parameters of a generalised F distribution. It was found that the original Wilshire-Scharning assumption of a constant activation energy for this materials is incorrect. Consequently, their interpretation of deformation being due only to dislocation creep with deteriorating microstructure at long duration test times appears to be ill founded, with the varying activation energy suggesting instead that deformation is due to grain boundary sliding accommodated by either dislocation and diffusional creep with dominance changing from the lattice to the grain boundaries as the temperature changes. Modelling batch to batch variation as a function of stress also resulted in a 50% extended safe life prediction (corresponding to a 1% chance of failure) at 873 K and 47 MPa.

## 1. Introduction

The prediction of long-term creep properties from short timescale experiments is rated as the most important challenge to the UK Energy Sector in a recent UK Energy Materials Review [1]. In response to this concern, a new approach—termed the Wilshire equations—has been devised which appears to allow accurate long-term strength values to be obtained by extrapolation from very accelerated short-term measurements. The last 5–6 years has seen the appearance in the literature of this methodology applied to a wide range of materials used for high temperature applications in the power generation and aerospace industries in an attempt to verify the validity and accuracy of this approach [2,3,4,5,6,7]. Specifically, 100,000 h strength estimates have been produced by analysis of multi-batch data lasting up to only 5000 h for a series of ferritic, bainitic and martensitic steels for power and petrochemical plants and titanium alloys for use in aero engine blades and disc.

The first aim of this paper is to illustrate how it is possible to further improve the accuracy of the Wilshire methodology by using iso-thermal plots to help identify changes in activation energies and the functional form for the Wilshire equation and to use these results to re-interpret the causes of deformation suggested by Wilshire and Scharning [3] for 1Cr-1Mo-0.25V. Such plots give insights into possible changes in creep regimes. The second aim is to show how to incorporate information on scatter present in creep data to obtain less conservative predictions of the time to failure associated with a particular risk of failure. To do this the paper is structured as follows. The next section briefly summarizes the data set on 1Cr-1Mo-0.25V steel used in this paper. Section 3 then briefly summarizes the Wilshire equation for time to failure and the findings made by Wilshire and Scharning [3] on this material. Section 4 re-analyses the data using iso-themal plots to identify a better specification for the Wilshire equation that allows for a change in the activation energy. Then, in Section 5 and Section 6 the two models are compared. Section 7 outlines a general way for incorporating batch to batch variation into the Wilshire equation and shows how it can be used to predict, not just the average time to failure, but also the whole shape of the failure time distribution. The final section provides conclusions and suggested areas for future work.

## 2. The Data

This present study features forged 1Cr-1Mo-0.25V steel for turbine rotors and shafts. For multiple batches of this bainitic product, both the creep and creep fracture properties have been documented comprehensively by the National Institute for Materials Science (NIMS), Japan [8]. NIMS creep data sheet No. 9B includes information on nine batches of as tempered 1Cr-1Mo-0.25V steel. Each batch of material had both a different chemical composition and a different thermal and processing history—details of which can be found in creep data sheet No. 9B. Specimens for the tensile and creep rupture tests were taken radially from the ring shaped samples which were removed from the turbine rotors. Each test specimen had a diameter of 10 mm with a gauge length of 50 mm.

These specimens were tested at constant load over a wide range of conditions: 333–47 MPa and 723–923 K. In addition to minimum creep rates (${\dot{e}}_{m}$) and times to failure (t_F_) measurements, the times to attain various strains (t_e_) at 0.005, 0.01, 0.02 and 0.05 over this range of test conditions were also presented. The values of the 0.2% proof stress (τ_Y_) and the ultimate tensile strength (τ_TS_) determined from high strain rate (~10^−3^ s^−1^) tensile tests carried out at the creep temperatures for each batch of steel investigated were also reported.

## 3. The Original Wilshire Study

The Wilshire equation for times to failure has the form,

(1) $$\left({\tau /\tau }_{\mathrm{TS}}\right)=\exp\{-k_{1}{\left[t_{F}\cdot \exp(-Q_{c}^{*}/\mathrm{RT})\right]}^{u}\}$$

where t_F_ is the time to failure, T is the absolute temperature, τ the stress, τ_TS_ the tensile strength, R the universal gas constant, Q^*^_c_ the activation energy for self-diffusion and k_1_ and u are further model parameters. This equation provides a sigmoidal data presentation such that t_F_ → ∞ as (σ/σ_TS_) → 0 whereas t_F_ → 0 as (σ/σ_TS_) → 1. Wilshire and Battenbough [2] proposed a very similar expression to Equation (1) for the stress and temperature dependencies of the minimum creep rate and the times to various different strains.

**Figure 1:**
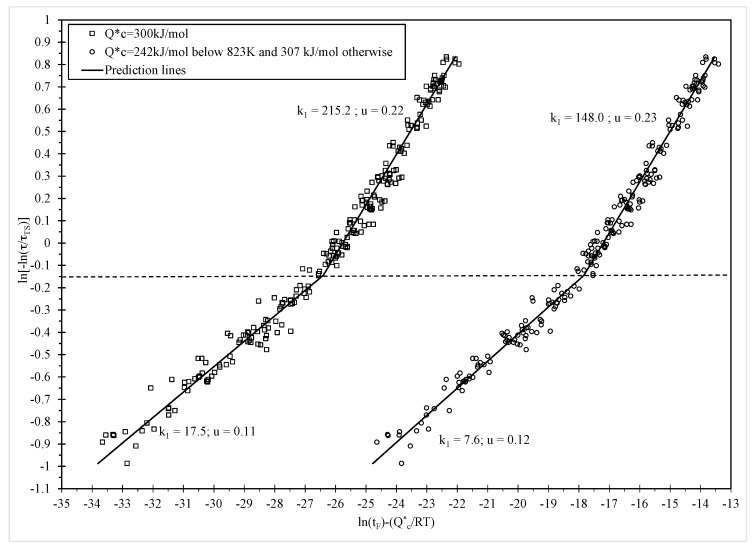
Dependence of ln[t_F_ exp(−Q^*^_c_/RT)] on ln[−ln(τ/τ_TS_)] at all temperatures with Q^*^_c_ = 300 kJ/mol (open squares) and Q^*^_c_ = 242 kJ/mol at or below 823 K, and Q^*^_c_ = 307 kJ/mol above 823 K (open circles).

In their original study on forged 1Cr-1Mo-0.25V steel for turbine rotors and shafts, Wilshire and Scharning [3] stated that Q^*^_c_ = 300 kJ·mol^−1^ and that this was the same at all stresses and temperatures. Under this assumption, the parameters k_1_ and u in Equation (1) appeared to be dependent upon stress and Figure 1 (open squares) replicates their findings with k_1_ and u changing abruptly at a normalised stress of about 0.42. The values for k_1_ and u shown in Figure 1 define the predicted values for the average time to failure.

The authors explained this observed change in the value for these parameter in terms of creep occurring predominantly through diffusion controlled generation and movement of dislocations within the lattice structure itself. Then the changes in k_1_ and u reflect differences in the rates of creep strength reduction caused by the evolution of the tempered bianitic microstructure in the low normalised stress range. This causes failure times to be much lower in the low stress regime than would be predicted by relations prevalent at higher stresses.

## 4. Re-Analysis of the Original Wilshire Study

The above description is not the only possible rationalisation for what is observed in Figure 1. The changing values for k_1_ and u could also be attributable to a change from creep occurring from the generation of new dislocations within the lattice structure itself to creep occurring from the movement of dislocations pre-existing in the grain boundary zones only. However, for this to be true, the activation energy would also need to change.

**Figure 2:**
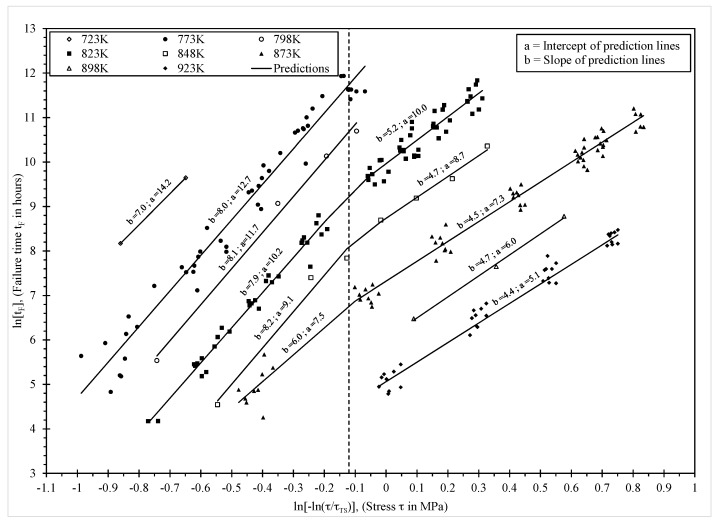
The temperature dependence of ln[t_F_ exp(−Q^*^_c_/RT)] on ln[−ln(τ/τ_TS_)]. Prediction lines are least squares best fit lines obtained separately for each temperature and for data above and below τ^*^ = −0.1.

The best way to investigate this possibility of a changing activation energy is to study the Wilshire equation at a given temperatures. In this respect, it is first helpful to rewrite Equation (1) in the following way

(2) $$\ln[t_{F}]=a+{b\tau }^{*}+Q_{c}^{*}\frac{1}{\mathrm{RT}}$$

with τ^*^ = ln[−ln(τ/τ_TS_)], b = 1/u, and a = ln[k_1_/u]. Then, at a constant temperature and a constant activation energy, $Q_{c}^{*}\frac{1}{\mathrm{RT}}$ becomes a constant and so can be “absorbed” into the intercept term a of Equation (2)

(3) $$\ln[t_{F}]=a+{b\tau }^{*}\;\mathrm{where}\;a=d+Q_{c}^{*}\frac{1}{\mathrm{RT}}$$

where the additional parameter d may or may not be zero in value. Thus, isothermal plots of ln[t_F_] against ln[−ln(τ/τ_TS_)] should define straight lines (with a kink at a normalised stress of about 0.42). With a constant activation energy, the effect of temperature is then to change the intercept but not the slope in Equation (3), so as to produce a series of parallel lines–one for each temperature. These isothermal Wilshire equations are shown in Figure 2. 

It appears that the data points are very different above and below τ^*^ ≈ −0.1. Thus, the solid lines in this figure are the least squares best fits to the data at each temperature, with a separate fit for when τ^*^ is above and below −0.1. Figure 2 also shows the resulting best fit values for a and b in Equation (3). Broadly speaking, these results suggest that below τ^*^ ≈ −0.1 the best fit lines shift down in a parallel fashion until a temperature of 873 K is reached. Then, with further temperature changes there is a non-parallel shift. Again, and broadly speaking, above τ^*^ ≈ −0.1, the best fit lines shift up in a parallel fashion until a temperature of 823 K is reached. Then with further temperature changes these is a non-parallel shift. These non-parallel shifts suggest the activation energy must be changing with respect to temperature.

**Figure 3:**
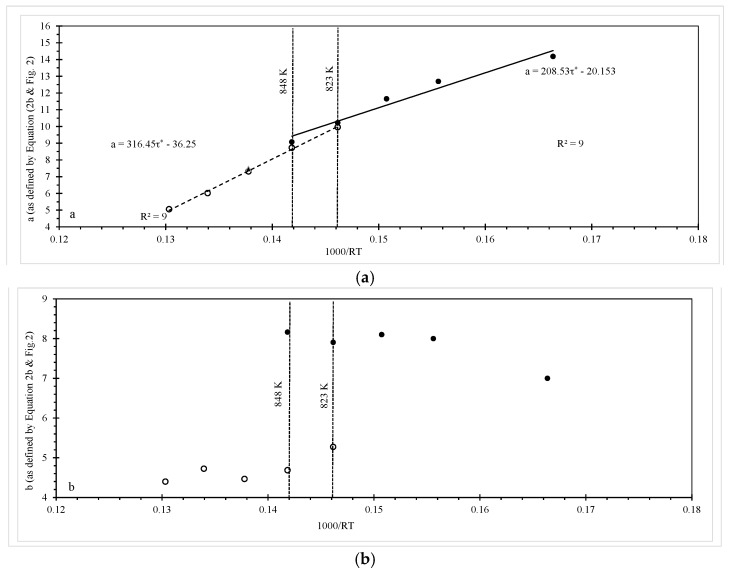
The dependence of: (**a**) the intercept a in Equation (3); and (**b**) the slope b in Equation (3) on the reciprocal of temperature.

This is further confirmed in Figure 3, where the slopes and intercept values shown in Figure 2 are plotted on the vertical axis of Figure 3. The reciprocal of temperature is then on the horizontal axis. In Figure 3 the solid circles correspond to the best fit slope and intercepts of Figure 2, but below τ^*^ ≈ −0.1, whilst the open circles correspond to the best fit slope and intercepts of Figure 2, but above τ^*^ ≈ −0.1. As Figure 3a plots the intercept on the vertical axis, the slopes of the best fit lines in this graph provided approximate estimates of the activation energy. Thus, somewhere between 823 K and 848 K the activation energy changes from around 209 kJ·mol^−1^ to round 317 kJ·mol^−1^. Notice that at 848 K and 823 K the value for a is estimated to be approximately the same using either the best fit lines above or below τ^*^ ≈ −0.1 in Figure 2. This suggests the activation energy is changing with respect to temperature and not the normalised stress. This is further confirmed by the single triangular data point of Figure 3a (at 873 K). This value is found from the best fit line at 873 K but when τ^*^ > −0.1. However, this a value is compatible with the open circles in Figure 3a and not the solid circles because the temperature change from 848 K to 873 K has resulted in a big change in the activation energy. Figure 3b reveals however that the best fit slopes in Figure 2 above and below τ^*^ ≈ −0.1 are relatively constant (around 4.5 when τ^*^ > −0.1 for example).

To summarise, the original Wilshire and Scharning study used the model

(4) $$\begin{array}{ll} \ln[t_{F}]=a_{1}+b_{1}\tau^{*}+300000\frac{1}{\mathrm{RT}} & \mathrm{when}\tau^{*}\leq \tau_{\mathrm{crit}}^{*}\\ \ln[t_{F}]=a_{2}+b_{2}\tau^{*}+300000\frac{1}{\mathrm{RT}} & \mathrm{when}\tau^{*}>\tau_{\mathrm{crit}}^{*} \end{array}$$

so that both the intercept and slope in the Wilshire equation changes at some critical value for the transformed normalised stress. In comparison to this, Figure 2 and Figure 3 suggest a more accurate representation of the data would be

(5) $$\begin{array}{ll} \ln[t_{F}]=a+b_{1}\tau^{*} & \mathrm{when}\tau^{*}\leq \tau_{\mathrm{crit}}^{*}\\ \ln[t_{F}]=a+b_{2}\tau^{*} & \mathrm{when}\tau^{*}>\tau_{\mathrm{crit}}^{*}\\ \mathrm{with} & \\ a=d_{1}+Q_{c}^{|*}\frac{1}{\mathrm{RT}} & \mathrm{when}T\leq T_{\mathrm{crit}}\\ a=d_{2}+Q_{c}^{|**}\frac{1}{\mathrm{RT}} & \mathrm{when}T>T_{\mathrm{crit}} \end{array}$$

where τ^*^_c_ and T_crit_ correspond to the transformed normalised stress and temperature at which there is a change in b and a change in the activation energy (from Q^*^_c_ to Q^**^_c_), respectively. That is, the slope b in this modified model changes at some critical value for the transformed normalised stress (as is the case in the original Wilshire equation), but the intercept of the Wilshire equation is now a linear function of the reciprocal of temperature. Further, this linear relationship changes at some critical temperature (which is not the case in the original Wilshire equation).

Based on this isothermal approach, the results shown in Figure 2 and Figure 3 suggest d_1_ = −20.15, d_2_ = −36.25, Q^*^_c_ = 209 kJ/mol and Q^**^_c_ = 317 kJ/mol. τ^*^_c_ and T_crit_ are around −0.1 and 823 K respectively. Values for b_1_ and b_2_ can be approximated by averaging the values shown in Figure 3b, assuming that the observed variation in b is random with respect to temperature. Then, b_1_ = 7.5 and b_2_ = 4.7.

## 5. A Comparison of the Original and Modified Wilshire Equations

It is important to realise that the above isothermal approach only gives approximate values for the unknown parameters in Equations (2) and (3). More accurate estimates can be obtained by first rewriting Equations (4) and (5) using a Max function. The original Wilshire equation given by Equation (4) can then be expressed as

(6) $$\ln[t_{F}]=a_{1}+b_{1}\tau^{*}+300000\frac{1}{\mathrm{RT}}+\Delta_{1}\mathrm{Max}[0,\tau^{*}-\tau_{\mathrm{crit}}^{*}]$$

where b_2_ = {b_1_ + Δ_1_}, a_2_ = {a_1_ − Δ_1_t^*^_crit_}, with Δ_1_ being a further parameter and Max[0,z] means return the larger of zero or z. The modified Wilshire equation given by Equation (5) then becomes

(7) $$\ln[t_{F}]=d_{1}+b_{1}\tau^{*}+Q_{c}^{*}\frac{1}{\mathrm{RT}}+\Delta_{1}\mathrm{Max}[0,\tau^{*}-\tau_{\mathrm{crit}}^{*}]+\Delta_{2}\mathrm{Max}[0,1/{\mathrm{RT}}_{\mathrm{crit}}-1/\mathrm{RT}]$$

where b_2_ = {b_1_ + Δ_1_}, d_2_ = {d_1_ − Δ_1_t^*^_crit_ + Δ_2_/RT_crit_}, Q^**^_c_ = {Q^**^_c_ − Δ_2_}, and Δ_2_ is a further parameter.

Fixing τ^*^_c_ and T_crit_ at particular values, enables values for ${\mathrm{Max}[0,\tau }^{*}-\tau_{\mathrm{crit}}^{*}]\mathrm{and}{\mathrm{Max}[0,1/\mathrm{RT}}_{\mathrm{crit}}-1/\mathrm{RT}]$ to be determined, so these can then be treated as separate variables in Equations (6) and (7). This allows multiple least squares to be used to obtain accurate estimates of all the unknown parameters. It is then possible to re-estimate these parameters for other values of τ^*^_c_ and T_crit_ in a grid search fashion. The best values for τ^*^_c_ and T_crit_ are then taken to be the ones that maximise the amount of explained variation in log failure times, i.e., the coefficient of determination or R^2^ value from the multiple regression (however, this approach does not allow for the estimation of the standard error associated with these cut off points). Table 1 summarises the results from applying this least squares grid search procedure to the original and modified Wilshire equation. 

All parameters are statistically different from zero at the 1% significance level and both equations explain around 98% of the variation in the log times to failure (as shown by the R^2^ value). For both equations a break occurs at around τ^*^ = −0.15, with a further change occurring in the activation energy at a temperature of around 823 K in the modified Wilshire equation. Using the estimated activation energies shown in Table 1 then allows the construction of the variable on the horizontal axis in Figure 1 and thus the data points shown in this figure. Substituting the parameter estimates shown in Table 1 into Equations (6) and (7) then produces the prediction lines shown in Figure 1 for each of the equations.

Table 1 reveals that the value for b_1_ and b_2_ in both the original Wilshire and modified Wilshire equations are quite similar and this explains why the values for u shown in Figure 1 are also very similar. Hence, for both equations, the prediction lines have a similar slope or shape. They are, however, separate along the horizontal axis because of the big difference between the activation energies used by each equation. This results in the values for the a and d parameters associated with each model being quite different and this results in the different k_1_ values shown in Figure 1. The results also show that the activation energy changes at a temperature of 823 K, with the activation energy being highest at the highest temperatures.

**Figure 4:**
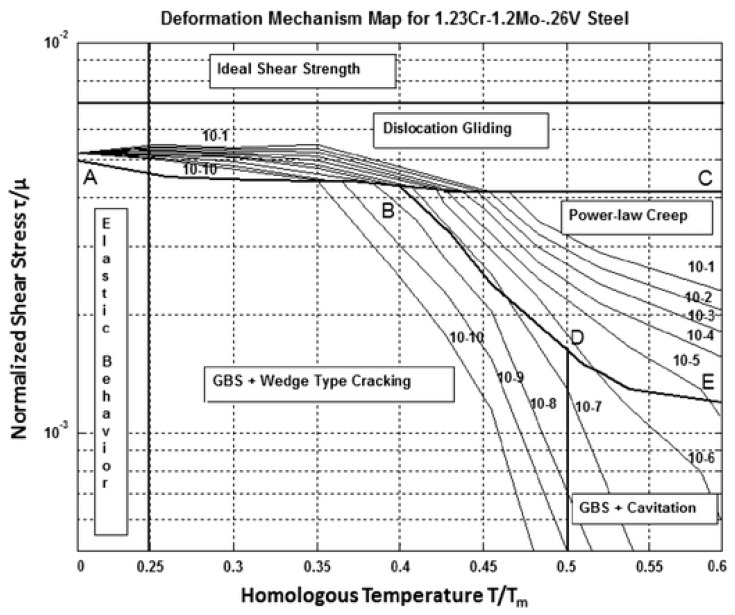
Deformation mechanism map produced by Bano et al. [9].

These results may reveal something important about creep deformation mechanisms as well. Wilshire and Scharning believed that the dominant creep mechanism under all test conditions was via diffusion controlled generation and movement of dislocations within the lattice structure itself and the kink observed in Figure 1 was then due the evolution of the tempered bianitic microstructure during prolonged testing in the low normalised stress range. However, this view does not explain the statistically significant and large change in activation energy with temperature observed in Table 1. An explanation for this change in activation energy can in part be aided by comparison to the deformation map, shown here in Figure 4, for this material provided by Bano et al. [9]. In doing so it must be remembered that the Wilshire equation is fundamentally different to such a map as this map is derived using different models and has mechanism changes that are continuous with respect to stress and temperature. In contrast, the Wilshire equation identified here has one abrupt change with respect to stress and one with respect to temperature. That said, at a temperature of 823 K or below, the vast majority of the NIMS tests fall into what Bano et al. call “grain boundary sliding with triple point cracking”. It is not clear from their paper whether this sliding is accommodated by grain deformation resulting from dislocation glide and climb within grain boundaries or vacancy flow along grain boundaries (Coble creep). Either way the activation energy would be lower than that for self-diffusion—hence the observed lower activation energy of Q^*^_c_ ≈ 240 kJ/mol. The activation energy would that associated with self-diffusion along grain boundaries. Some further light can be shed on this by looking at the NIMS data above 823 K. Here the vast majority of the NIMS tests fall into either what Bano et al. call “grain boundary sliding with triple point cracking” (60% of NIMS data are in this Bano et al. region) or “grain boundary sliding with cavitation’s” (40%). Again, it is not clear from the Bano et al. paper whether this sliding with cavitation is accommodated by grain deformation resulting from dislocation glide and climb within grain boundaries, or vacancy flow along grain boundaries (Coble creep), or vacancy flow within grain boundaries (Nabarro–Herring creep). However, the only way to explain the observed higher activation energy of Q^*^_c_ ≈ 300 kJ/mol at these higher temperatures within the NIMS data is to take it as Nabarro–Herring creep. The measured activation energy would then be higher than for the low temperature NIMS data but not quite that for self-diffusion as a big proportion of the data is within the grain boundary sliding region with triple point cracking.

This would then explain why b_1_ (that summarizes the stress relationship) in Table 1 changes above and below t^*^_crit_. At or below 823 K the vast majority of the NIMS data is also below t^*^_crit_ and so corresponds to b_1_ = 8.234 in Table 1. Then above 823 K the vast majority of the NIMS data is also above t^*^_crit_ and so corresponds to b_1_ = 4.392 in Table 1. This is a change in the relationship between t_F_ and stress similar to a change in the value for Norton’s n in the power law equation. The only way to explain this change in b_1_ as temperature changes, and remain compatible with the view expressed in the paragraph above, is to take the Bano et al. grain boundary sliding region with triple point cracking to be accommodated by grain deformation resulting from dislocation glide and climb within grain boundaries as then there would be a change in Norton’s n and so a change in b_1_ (as well as the observed change in the activation energy).

## 6. Predicted Time to Failure

**Figure 5:**
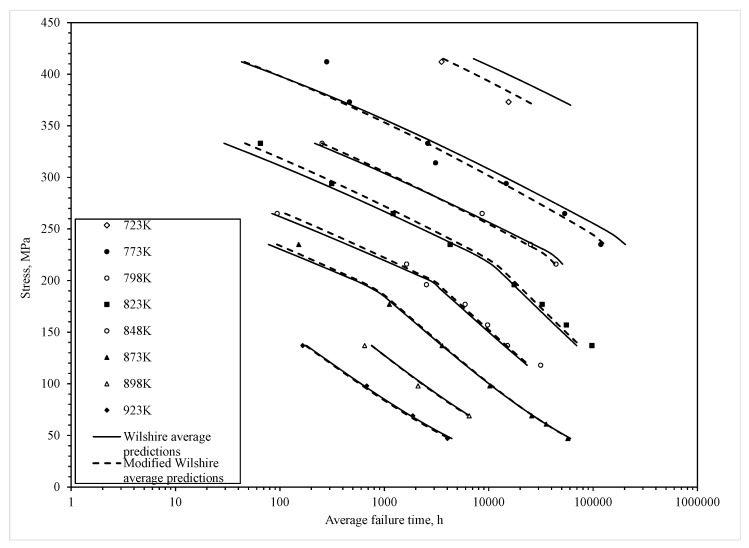
The stress dependence of average (over all batches) times to failure compared to predicted curves from the original (Equation (4)) and modified Wilshire (Equation (5)) equations.

It is not clear from Figure 1 how good each model is at predicting the recorded times to failure because of the temperature compensated transformation of the failure times shown on the horizontal axis. This is best seen by plotting the data in the more familiar stress/failure time space. In the NIMS data base, a number of different batches of steel are tested at various stress–temperature combinations. The number of batches tested varies with this test combination but is typically between 2 to 9 batches. Thus, in Figure 5, the average time to failure over all batches tested at these different stress–temperature combinations is shown.

Figure 5 shows the average predictions obtained from Equations (6) and (7) using the parameter estimates shown in Table 1. To get the average prediction out from each equation a separate prediction was made for each batch at each stress–temperature combination. These batch predictions were then averaged. The resulting average predictions from each of the equations is then shown in Figure 5. Over all, it is clear that the modified Wilshire equation is much more capable of predicting the average time to failure. This is especially noticeable at the lower stress associated with a temperature of 773 K. Here, the original Wilshire equation dramatically over estimates the average time to failure as it also does at 723 K. The modified Wilshire equation also performs significantly better at the higher stress associated with a temperature of 823 K.

## 7. Batch to Batch Variation and the Wilshire Equation

Figure 5 does not say anything about how well each model predicts the times at which individual batches fail. For engineering companies using a particular batch of steel this information is important to them in obtaining a safe life for their operations. To be able to assess how well each Wilshire equation does in batch to batch predictions, a failure time distribution needs to be specified and this in turn requires an estimation approach different to least squares.

### 7.1. A Generalised Failure Time Distribution 

One generalised linear model for ln(t_F_) takes the form

(8) $${\ln[t}_{F}]=\mu +\sigma v$$

where μ is a scaling parameter which in this application is given by

(9) $$\mu =d_{1}+b_{1}\tau^{*}+Q_{c}^{*}\frac{1}{\mathrm{RT}}+\Delta_{1}\mathrm{Max}[0,\tau^{*}-\tau_{\mathrm{crit}}^{*}]+\Delta_{2}\mathrm{Max}[0,1/{\mathrm{RT}}_{\mathrm{crit}}-1/\mathrm{RT}]$$

and σ is a shape parameter so that the random variable v can be interpreted as following a standardised distribution. v is obviously incorporated into the model to pick up the batch to batch variation present in the experimental data. As the nature of the creep failure time distribution is generally unknown, it makes sense to select the most general possible representation of this distribution. On such distribution is the generalised F distribution. Therefore, in this paper, the variable v is taken to be the logarithm of an F variate with 2κ_1_ and 2κ_2_ degrees of freedom. The probability density function (PDF) for v is then given by

(10) $$f(v)=\frac{{\left(\kappa_{1}/\kappa_{2}\right)}^{k_{1}}}{\left\{\Gamma (\kappa_{1})\Gamma (\kappa_{2})\right\}/{\Gamma (\kappa }_{1}+\kappa_{2})}\frac{\exp(\kappa_{1}v)}{{\left(1+\left\{\kappa_{1}/\kappa_{2}\right\}e^{v}\right)}^{\left(\kappa_{1}+\kappa_{2}\right)}}$$

where Γ() is the gamma function and μ and σ, together with κ_1_ and κ_2_, are the parameters of this four parameter log F distribution. Values of (κ_1_, κ_2_) equal to (1, 1), (1, ∞) and (∞, 1) correspond, respectively, to the logistic, extreme value for a minima and extreme value for a maxima for ln[t_F_]. As κ_2_ approaches infinity the distribution for v approaches the logarithm of a generalised gamma variate as presented by Stacy and Mihram [10]. When σ = 1 the familiar gamma distribution is obtained. When (κ_1_ = ∞, κ_2_ = ∞) the distribution for ln[t_F_] corresponds to a normal distribution (and so the variable v becomes the standard normal distribution). However, as specified in Equation (10), the distribution is degenerate in nature. Prentice [11] has shown that such degeneracy can be avoided by transforming v in the following way

(11) $$v=\sqrt{\kappa_{1}\kappa_{2}/[\kappa_{1}+\kappa_{2}]}w=\delta w$$

so that

(12) $$\ln[t_{F}]=\mu +\sigma \delta w=\mu +\eta w\;\mathrm{or}\;w=\frac{{\ln[t}_{F}]-\mu }{\eta }$$

The probability density function (PDF) for w can be found by substituting Equation (11) into Equation (10)

(13) $$f(w)=\frac{{\left(\kappa_{1}/\kappa_{2}\right)}^{\kappa_{1}}}{\left\{\Gamma (\kappa_{1})\Gamma (\kappa_{2})\}/\Gamma (\kappa_{1}+\kappa_{2}\right)}\frac{\exp(\kappa_{1}\delta w)}{{\left(1+\{\kappa_{1}/\kappa_{2}\}e^{\delta w}\right)}^{\left(\kappa_{1}+\kappa_{2}\right)}}$$

At κ_1_ = κ_2_ = ∞, w has a standard normal distribution. The probability density function (PDF) for y = ln[t_F_] is

(14) $$f(y)=\frac{\delta {\left(\kappa_{1}/\kappa_{2}\right)}^{\kappa_{1}}}{\eta \{\Gamma (\kappa_{1})\Gamma (\kappa_{2})\}/\Gamma (\kappa_{1}+\kappa_{2})}\frac{\exp(\kappa_{1}\delta w)}{{\left(1+\{\kappa_{1}/\kappa_{2}\}e^{\delta w}\right)}^{\left(\kappa_{1}+\kappa_{2}\right)}}$$

Values of (κ_1_, κ_2_) equal to (1, 1), (1, ∞), (∞, 1) and (∞, ∞) correspond, respectively, to the logistic, extreme value for a minima, extreme value for a maxima and normal distributions for ln[t_F_]. Consequently, values of (κ_1_, κ_2_) equal to (1, 1) and (∞, ∞) correspond, respectively, to the log-logistic and (non degenerate) log normal distribution for t_F_. The Weibull distribution for t_F_ is obtained at (κ_1_, κ_2_) = (1, ∞). If in addition η = 1, the exponential distribution for t_F_ is obtained. The generalised gamma distribution corresponds to κ_2_ = ∞ and this reduces to the gamma distribution for η = 1. 

The distribution for ln[t_F_] corresponding to κ_2_ = ∞ is essentially the logarithm of a generalised gamma variate which has recently been applied by Evans [12] to high temperature life time data. The PDF given by Equation (14) is positively skewed for κ_1_ > κ_2_, negatively skewed for κ_1_ < κ_2_ and symmetric for κ_1_ = κ_2_.

Within this framework, it is also easy to allow for the variability in failure times to depend on stress, which is a phenomenon often seen in large multi batch uniaxial creep test programs. One possible specification for this stress dependant heteroscedasticity that does not allow for a negative variance at any stress level is

(15) $$\eta =e^{\beta_{0}+\beta_{1}\tau^{*}}$$

so that β_1_ = 0 corresponds to homoscedasticity or constant variability in failure times, which is the implicit assumption of the original Wilshire specification. The rth percentile time to failure is then given by

(16) $$t_{F,r}=\exp\{\mu +{\eta w}_{\kappa 1,\kappa 2,r}\}$$

where w_k1,k2,r_ is the rth percentile of the logarithm of an F distributed variate with 2κ_1_ and 2κ_2_ degrees of freedom.

### 7.2. Parameter Estimation

The parameters μ, η, κ_1_ and κ_2_ can be estimated from a sample of observations on t_F_. Suppose that a sample of n log (natural) times to failure have been collected. Assuming that these observations are independent, the probability of actually observing this sample of log times to failure is given by Equation (14) and the product rule of probability,

(17) $$L(\mu ,\eta ,\kappa_{1}\kappa_{2})=\frac{\delta {\left(\kappa_{1}/\kappa_{2}\right)}^{\kappa_{1}}}{\eta \left\{\Gamma (\kappa_{1})\Gamma (\kappa_{2})\right\}/\Gamma (\iota_{1}+\kappa_{2})}\overset{n}{\underset{i=1}{\Pi }}\frac{\exp(\kappa_{1}{\delta w}_{i})}{{\left(1+\{\kappa_{1}/\kappa_{2}\}e^{{\delta w}_{i}}\right)}^{\left(\kappa_{1}+\kappa_{2}\right)}}$$

where L(μ, η, κ_1_, κ_2_) is called the likelihood of observing the sample of log times to failure, which of course depends on the values for μ, η, κ_1_, κ_2_. Within this framework, it makes sense to choose values for μ, η, κ_1_ and κ_2_ that maximise this likelihood. Because it is often easier to work with sums rather than products, values for μ, η, κ_1_ and κ_2_, are in practice chosen to maximise the log likelihood, ln L(μ, η, κ_1_, κ_2_).

The values for μ, η, κ_1_ and κ_2_ that maximise ln L(μ, η, κ_1_, κ_2_) are called maximum likelihood estimates and are given the symbols $\;\tilde{\mu },\tilde{\eta },{\tilde{\kappa }}_{1}\;\mathrm{and}\;{\tilde{\kappa }}_{2}$. Maximising the log of Equation (17) requires simultaneously solving the equations $\partial \mathrm{lnL}(\mu ,\eta ,\kappa_{1},\kappa_{2})/\partial \mu =0$, $\partial \mathrm{lnL}(\mu ,\eta ,\kappa_{1},\kappa_{2})/\partial \eta =0$; $\partial \mathrm{lnL}(\mu ,\eta ,\kappa_{1},\kappa_{2})/\partial \kappa_{1}=0$, $\partial \mathrm{lnL}(\mu ,\eta ,\kappa_{1},\kappa_{2})/\partial \kappa_{2}=0$.

As Equation (17) stands, finding such a solution is rather difficult because these partial derivatives are not finite and in some cases are identically zero along the boundaries κ_1_ = ∞ and κ_2_ = ∞. This makes it all the more difficult to discriminate between various distributions for ln[t_F_]—especially the log-normal distribution. As shown by Prentice [11], the following re-parameterisation leads to a maximised log likelihood function with regular (finite and not identically zero) likelihood derivatives everywhere on the boundary κ_1_ = ∞ and κ_2_ = ∞

(18) $$p=2{\kappa_{1}+\kappa_{2}}^{-1}\;\mathrm{and}\;q=(\kappa_{1}^{-1}-\kappa_{2}^{-1})({\left(\kappa_{1}^{-1}-\kappa_{2}^{-1}\right)}^{-1/2}$$

Under this parameterisation, the log-normal, Weibull, log-logistic, reciprocal Weibull, and generalised gamma distributions for t_F_ occur, respectively, at (q, p) values of (0, 0), (1, 0), (0, 1), (−1, 0) and (q > 0, 0). The maximum likelihood solution can be further simplified by using numerical rather than analytical derivatives and by treating q and p as fixed, so that maximisation of the log of Equation (9) only requires simultaneously solving the equations

(19) ∂lnL(μ,η,q,p)/∂μ=0, ∂lnL(μ,η,q,p)/∂η=0

As shown by Lawless [13], this implies a simple two-step grid search procedure. For a given value of p and q and τ^*^_c_ and T_crit_ in Equations (9), (12), (17) and (18), first find the values $\tilde{\mu }(q,p)\;\mathrm{and}\;\tilde{\eta }(q,p)$ that maximise ln L(μ, η, q, p) by solving the above equations with the specified values for q, p and τ^*^_c_, T_crit_. Secondly, if this is done for various values of q, p, τ^*^_c_ and T_crit_ the largest of all these maximised log likelihood’s, termed the grand maximised log likelihood, can be obtained and so q, p, τ^*^_c_ and T_crit_ located. Berndt et al. [14] give details of how these derivatives can be solved numerically.

### 7.3. Findings

**Figure 6:**
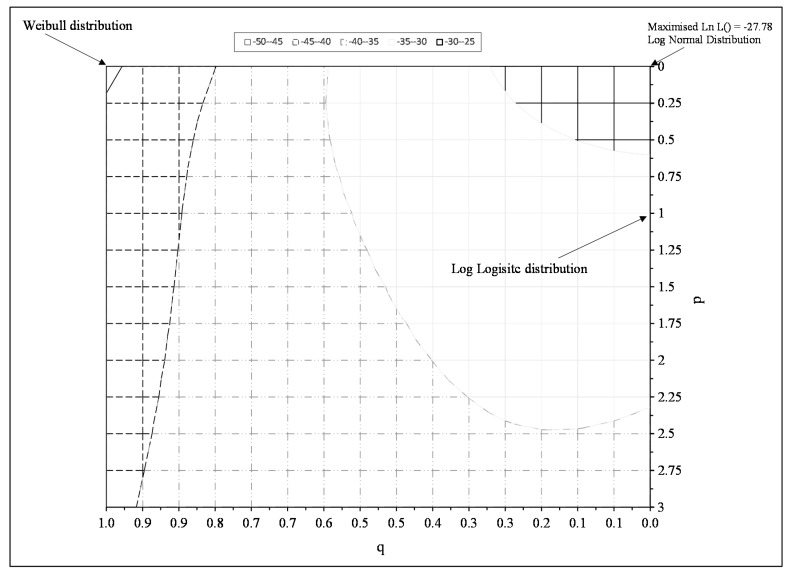
The maximised log likelihood function given by the log of Equation (17) obtained at differing values for p and q.

Figure 6 shows the results of the above grid search procedure and the ln of the likelihood function is maximised when p = q = 0, suggesting that the normal distribution for ln[t_F_] best describes all the experimental failure times.

**Figure 7:**
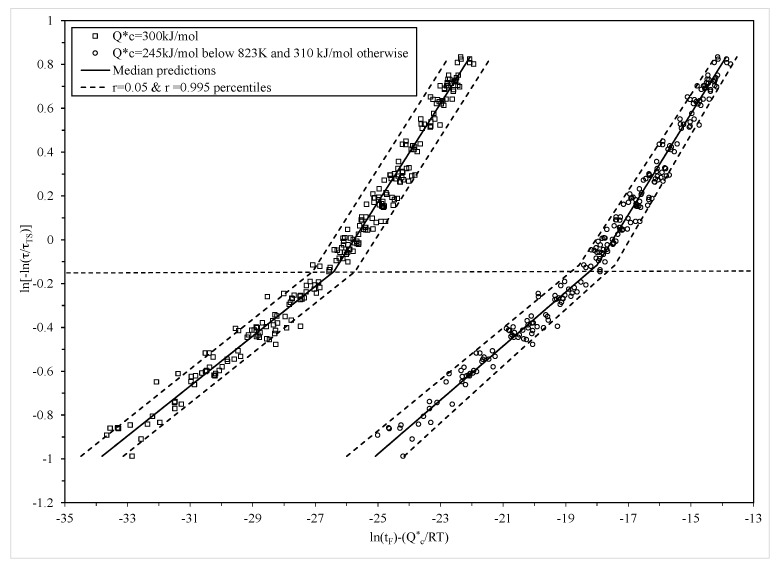
Dependence of ln[t_F_ exp(−Q^*^_c_/RT)] on ln[−ln(τ/τ_TS_)] at all temperatures with Q^*^_c_ = 310 kJ/mol (open squares) and with Q^*^_c_ = 244 kJ/mol below 823 K and Q^*^_c_ = 307 kJ/mol above 823 K (open circles), together with 0.05 and 0.995 percentiles from the normal distribution.

The maximum likelihood estimates for the unknown parameters in Equations (9), (15) and (18), associated with this grand maximised log likelihood, are shown in Table 2. The parameter estimates in Table 2 are similar to those in Table 1 and do not alter the interpretations made earlier about creep mechanisms. However, the statistical significance of β_1_ means that the variability in the experimental times to failure increases with increasing stress levels. This is clearly seen in Figure 7, where the r = 0.05 and r = 0.995 percentiles of the time to failure are shown and are based on Equation (16). This produced a 99% prediction band for times to failure around the median (r = 0.5 percentile) time to failure shown as the solid segmented line in Figure 7. In this figure, the left most plot of data are the results from applying the traditional Wilshire equation, based on the parameters in Table 1, with a constant failure time variance and a normal distribution. The constant variance is seen by the constant width of the prediction bands. At the higher stress levels, this specification does not work well with more data points falling outside this band compared to at the lower stress levels. That is, the bands are too wide at lower stresses and to narrow at the higher stresses.

The plot of data to the right is the modified Wilshire equation based on the parameters in Table 2 that allows for non-constant variance within the normal distribution. It can now be seen that very few data points are outside this prediction band, and the narrower width of the band at the lower stresses allows for less conservative safe life estimates to be made for this material, whilst still maintaining a less than 0.05% chance of failure before this safe life prediction.

**Figure 8:**
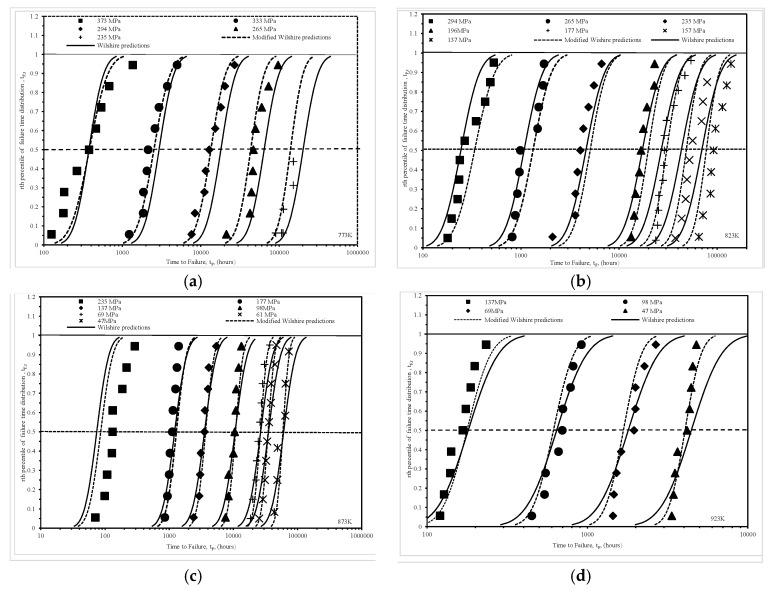
Empirically estimated probabilities to failure together with predicted probabilities obtained from the original and modified Wilshire equations at various stresses and temperatures of (**a**) 7733 K, (**b**) 823 K; (**c**) 873 K and (**d**) 923 K.

Figure 8 presents another way of comparing how well these two approaches do at predicting various percentiles of the time to failure distribution. In this figure the shown data points are the empirical percentiles as they are calculated only from the failure times themselves using

(20) $$t_{F,r}^{E}=\frac{i-0.5}{n}$$

and as such make no distributional assumptions in their derivation. In Equation (20), n represents the number of failed specimens obtained at a particular stress–temperature combination and i is a rank index—equal to 1 for the smallest recorded failure time through to n for the largest recorded failure time at this test condition.

In Figure 8, these empirical percentiles are plotted against the actual failure times at various stress–temperature combination to yield the symbol data points shown. The curves then show the rth percentile failure predictions obtained using the traditional Wilshire and modified Wilshire approaches. Highlighted with a horizontal line is the median actual and predicted time to failure at each of the stress–temperature combinations. At 773 K, it is clear that the modified approach yields better median time to failure predictions at all the different stress levels and, whilst still not perfect, it also yields better predictions at the tail ends of the failure time distribution as well.

The same picture emerges at 873 K, although both techniques fail to predict any percentile time to failure at the highest stress of 235 MPa. At 923 K, the modified approach clearly predicts the shape of the actual failure time distribution much better than the traditional Wilshire approach, but at the highest stress neither approach has worked particularly well—even though the median predicted time to failure is reasonable. Finally, at 823 K although neither approach describes the failure time distribution well at the very lowest stresses, the modified approach is the better of the two.

## 8. Conclusions

This paper has demonstrated that the use of isothermal plots of the Wilshire equation can identify better functional forms for the relationship between the log time to failure and transformations of stress and temperature. In particular, these plots revealed for the first time that, for 1Cr-1Mo-0.25V steel, the activation energy increases with temperature—changing from around 240 kJ/mol to around 300 kJ/mol at a temperature somewhere between 823 K and 848 K. This result challenges the original Wilshire-Scharning explanation for creep deformation in this material. Instead of deformation being due only to dislocation creep with deteriorating microstructure at long duration test times, the varying activation energy suggests this deformation is due to both dislocation and diffusional creep (with grain boundary sliding). At high stresses and temperatures, there is grain boundary sliding with caviation accommodated by vacancy flow within grain boundaries (Nabarro-Herring creep) with dominance changing to grain boundary sliding with triple point cracking accommodated by grain deformation resulting from dislocation glide and climb within grain boundaries. 

The use of a variable activation energy within the Wilshire equation enabled more accurate predictions to be made of the average and median times to failure—especially at 723 K, 773 K and 823 K. Then, through the use of a generalised F distribution, this paper further demonstrated that the batch to batch variation in creep failure times for this material are best described through use of the log normal distribution—rather than any other member of the exponential distribution family. This paper also demonstrated that the variation in times to failure increased with increasing stress, and building this phenomenon into the Wilshire equation enabled much less conservative times to failure to be predicted at the lower stresses that correspond more closely to operating conditions for this material. For example, the traditional Wilshire equations with a normal distribution predicts that replacement should be at three years to reduce the chances of failure in use at 873 K and 47 MPa to 1% or less. The corresponding value based on the modified equations that allows for heteroscedasticity puts the replacement time at 4.5 years for the same level of risk (in both these illustrations the average recorded failure time is 6.5 years).

It would be of interest to see if the model building approach identified in this paper alters the conclusions already made using the Wilshire equations on other high temperature materials. Whilst the distributional approach used in this paper works well at most test conditions, there are still large prediction errors being made at some test conditions implying another area for future research.

## Figures and Tables

**Table 1:** Least squares estimates of the parameters in Equations (4)–(7).

Parameter	Original Wilshire Equation: Equations (4) and (6)		Modified Wilshire Equation: Equation (5) and (7)	
	Estimate	Student t	Estimate	Student t
a_1_	−25.135	−431.03 ***	-	-
b_1_	8.786	66.19 ***	8.234	64.29 ***
Δ_1_	−4.343	−22.30 ***	−3.841	−21.95 ***
a_2_	−23.864	−368.45 ***	-	-
b_2_	4.443	18.85 ***	4.392	20.25 ***
d_1_	-	-	−16.646	−18.63 ***
Q^*^_c_ (kJ/mol)	300	-	242.029	39.73 ***
Δ_2_	-	-	−64.838	−8.21 ***
d_2_	-	-	−26.678	−18.27 ***
Q^**^_c_	-	-	306.867	30.76 ***
τ^*^_crit_	−0.145	-	−0.145	-
T_crit_ (K)	-	-	823	-
R^2^ (%)	98.56	-	98.02	-

Student t tests the null hypothesis that the true parameter values equal zero. * identifies statistically significant variables at the 10% significance level, ** identifies statistically significant variables at the 5% significance level, and *** identifies statistically significant variables at the 1% significance level. R^2^ is the coefficient of determination adjusted for the degrees of freedom. - indicates variables not in the specified model or student t value not available due to it being treated as a fixed value in the grid search procedure.

**Table 2:** Maximum likelihood estimates of the parameters in Equations (9), (15) and (18).

Parameter	Modified Wilshire Equation	
	Estimate	Student t
a_1_	-	-
b_1_	8.115	51.92 ***
Δ_1_	–3.774	–19.56 ***
b_2_	4.342	17.49 ***
d_1_	–17.067	–15.17 ***
Q^*^_c_ (kJ/mol)	244.381	31.74 ***
Δ_2_	–65.175	–7.13 ***
d_2_	–27.025	–15.47 ***
Q^**^_c_	309.556	25.89 ***
β_0_	–1.292	–24.21 ***
β_1_	–0.538	–4.55 ***
τ^*^_crit_	–0.115	-
T_crit_ (K)	823	-
p,q	0,0	-
Ln L()	–27.778	-

Student *t* tests the null hypothesis that the true parameter values equal zero.* identifies statistically significant variables at the 10% significance level, ** identifies statistically significant variables at the 5% significance level, and *** identifies statistically significant variables at the 1% significance level. Ln L() is the grand maximised log likelihood given by the log of Equation (17). - indicates variables not in the specified model or student t value not available due to it being treated as a fixed value in a grid search.
